# Granulomatous-Lymphocytic Interstitial Lung Disease in Common Variable Immunodeficiency—Features of CT and ^18^F-FDG Positron Emission Tomography/CT in Clinically Progressive Disease

**DOI:** 10.3389/fimmu.2020.617985

**Published:** 2021-01-26

**Authors:** Mai Sasaki Aanensen Fraz, Natasha Moe, Mona-Elisabeth Revheim, Maria L. Stavrinou, Michael T. Durheim, Ingvild Nordøy, Magnhild Eide Macpherson, Pål Aukrust, Silje Fjellgård Jørgensen, Trond Mogens Aaløkken, Børre Fevang

**Affiliations:** ^1^ Section of Clinical Immunology and Infectious Diseases, Oslo University Hospital, Oslo, Norway; ^2^ Division of Radiology and Nuclear Medicine, Oslo University Hospital, Oslo, Norway; ^3^ Institute of Clinical Medicine, University of Oslo, Oslo, Norway; ^4^ Department of Respiratory Medicine, Oslo University Hospital, Oslo, Norway; ^5^ Research Institute of Internal Medicine, Oslo University Hospital, Oslo, Norway; ^6^ Centre for Rare Diseases, Oslo University Hospital, Oslo, Norway

**Keywords:** GLILD, Interstitial lung disease (ILD), Primary immumunodeficiencies, DLCO, rituximab, CVID- Common Variable Immunodeficiency Disorders, Pulmonary CT, FDG – PET

## Abstract

Common variable immunodeficiency (CVID) is characterized not only by recurrent bacterial infections, but also autoimmune and inflammatory complications including interstitial lung disease (ILD), referred to as granulomatous-lymphocytic interstitial lung disease (GLILD). Some patients with GLILD have waxing and waning radiologic findings, but preserved pulmonary function, while others progress to end-stage respiratory failure. We reviewed 32 patients with radiological features of GLILD from our Norwegian cohort of CVID patients, including four patients with possible monogenic defects. Nineteen had deteriorating lung function over time, and 13 had stable lung function, as determined by pulmonary function testing of forced vital capacity (FVC), and diffusion capacity of carbon monoxide (DLCO). The overall co-existence of other non-infectious complications was high in our cohort, but the prevalence of these was similar in the two groups. Laboratory findings such as immunoglobulin levels and T- and B-cell subpopulations were also similar in the progressive and stable GLILD patients. Thoracic computer tomography (CT) scans were systematically evaluated and scored for radiologic features of GLILD in all pulmonary segments. Pathologic features were seen in all pulmonary segments, with traction bronchiectasis as the most prominent finding. Patients with progressive disease had significantly higher overall score of pathologic features compared to patients with stable disease, most notably traction bronchiectasis and interlobular septal thickening. 18F-2-fluoro-2-deoxy-D-glucose (^18^F-FDG) positron emission tomography/CT (PET/CT) was performed in 17 (11 with progressive and six with stable clinical disease) of the 32 patients and analyzed by quantitative evaluation. Patients with progressive disease had significantly higher mean standardized uptake value (SUVmean), metabolic lung volume (MLV) and total lung glycolysis (TLG) as compared to patients with stable disease. Nine patients had received treatment with rituximab for GLILD. There was significant improvement in pathologic features on CT-scans after treatment while there was a variable effect on FVC and DLCO.

**Conclusion:**

Patients with progressive GLILD as defined by deteriorating pulmonary function had significantly greater pathology on pulmonary CT and FDG-PET CT scans as compared to patients with stable disease, with traction bronchiectasis and interlobular septal thickening as prominent features.

## Introduction

Common variable immunodeficiency (CVID) is the most common symptomatic primary immunodeficiency in adults with a prevalence of 1:50,000–1:25,000 in Caucasians (1). Patients are characterized by decreased levels of immunoglobulin (Ig) G, IgA, and/or IgM, typically resulting in recurrent respiratory infections with encapsulated bacteria (2). Up to 70% of CVID patients also present with non-infectious inflammatory complications (3). Interstitial lung disease (ILD) is a common non-infectious manifestation of CVID, and is associated with increased morbidity and mortality (4). The clinical picture ranges from asymptomatic patients with radiological ILD features only, to patients with chronic respiratory failure in need of lung transplantation. The natural disease course is variable, and there are few known early predictors of a progressive disease course.

The term “granulomatous-lymphocytic interstitial lung disease (GLILD)” was first proposed in 2004 by Bates et al. (4). They categorized a group of CVID patients as having GLILD after histological findings in lung biopsies that included granulomas, lymphoid interstitial pneumonitis, lymphoid hyperplasia, and follicular bronchiolitis. Others have described an even broader and combined pathological spectrum in CVID patients with ILD, with histological findings also including organizing pneumonia, non-specific interstitial pneumonia, and diffuse lymphoid hyperplasia (5–7). These findings could represent variation within a spectrum of benign lymphoproliferative lung pathology, or several different pathophysiological mechanisms (5, 6, 8). However, the need for lung biopsies in GLILD diagnosis is debated (9), and the need for other diagnostic tools with less risk of complications is clearly warranted.

Radiologically, GLILD has been characterized by CT findings such as reticulation, bronchial wall thickening, pulmonary nodules, and ground glass opacities, and CT is widely used in the management of these patients (10, 11). FDG-PET/CT imaging is a promising approach in the evaluation of inflammatory disease and has been reported in case studies of GLILD, but has not been evaluated in a larger cohort (12, 13).

Systemic corticosteroids are considered as first-line treatment in patients with GLILD, but the evidence to support this is limited (9). Rituximab alone or in combination with azathioprine or mycophenolate has been reported effective in some retrospective studies and case reports (7, 13–17). There are also case reports describing positive effects of sirolimus, TNF-inhibitors, methotrexate, hydroxychloroquine, cyclosporine, and mycophenolate alone (18, 19). However, there is no consensus regarding optimal treatment of this disorder and no randomized studies have been performed.

We aimed to further elucidate the roles of non-invasive diagnostic tools in GLILD, and in this retrospective observational study we present clinical, immunological, and radiological (including both CT and FDG PET/CT) features in our cohort of patients with GLILD. We compare these features in patients with stable or progressive clinical disease based on functional pulmonary testing. We also describe lung function trajectory and changes in CT and FDG-PET/CT findings among patients treated with rituximab.

## Methods

### Patient Population

Patients were recruited from a cohort of 240 CVID patients that are or have been followed at of the Section of Clinical Immunology and Infectious Diseases at Oslo University Hospital. CVID was defined as having decreased serum levels of IgG, IgA, and/or IgM by a minimum of two standard deviations below the mean for age, while excluding other causes of hypogammaglobulinemia. Written informed consent was obtained from all included patients and the study was approved by the Regional Ethical Committee (REC South-Eastern Norway, no 2012/521 and 33256). Patients with pulmonary CT descriptions suggestive of ILD and/or GLILD in a retrospective screening of their electronic medical record were included.

### Clinical and Laboratory Data

Laboratory and clinical data, including data on immunomodulatory treatment, were collected by retrospective review of electronic medical records. The patients’ most recent laboratory data for lymphocyte profile with B- and T-cell subpopulations were registered, and where possible, IgA-, IgM- and IgG-levels measured at the same time point. In patients who had received rituximab or other immunomodulatory treatment for GLILD, the most recent laboratory data prior to this treatment was chosen. In patients receiving intravenous immunoglobulins, immunoglobulins were measured immediately prior to infusion.

### Pulmonary Function Tests and Definition of Stable and Progressive Disease

All pulmonary function test (PFT) results including forced vital capacity (FVC) and diffusing capacity for carbon monoxide (DLCO) performed at our clinic from the patient’s first visit until April 2020 were registered. By assessing the change over time in pulmonary function tests, we defined a group with progressive GLILD. These had an absolute decline in FVC percent predicted > 10 percentage points (p.p.) and/or DLCO percent predicted >15 p.p. during the follow-up period. Patients who already had FVC percent predicted < 50 and/or DLCO percent predicted < 40 at their first PFT performed at our hospital were also included in this group, as the decline in lung function was assumed to have started prior to follow-up at our hospital. Patients not meeting these criteria for progressive disease were defined as stable.

The patients treated with rituximab were categorized by pre-treatment DLCO percent predicted above or below 55%, a cut-off derived from the ILD-GAP model, a scoring tool that has shown to perform well in predicting mortality in patients with chronic ILD (20).

### CT Imaging

We examined the most recent HRCT performed in each of the 32 patients, if possible avoiding CT performed during acute lower airway infections or when the patient received immunomodulatory therapy for any reason. In patients treated with rituximab targeting GLILD we examined the last CT prior to treatment, and also the first CT after the initial dose of rituximab (ranging from 3 to 16 months after the initial dose). The images were reviewed in consensus on a PACS (Picture Archiving and Communication System) screen in random order by two experienced chest radiologists, blinded to the patients´ lung function and clinical condition. All CT examinations except one were done at our institution.

Thin-section CT images were obtained in the supine position during breath-holding and deep inspiration. Supplementary expiratory scans were obtained in nine patients to verify small airways disease. For evaluation of the lung parenchyma and airways we applied thin reconstructed slice thickness (0.9–1.25 mm) with a high-spatial-frequency hard kernel, 2.5 mm contiguous images in the axial, coronal, and sagittal planes were in addition reconstructed with a medium soft algorithm. Tube current settings were adjusted to each patient’s weight.

**Figure 1:**
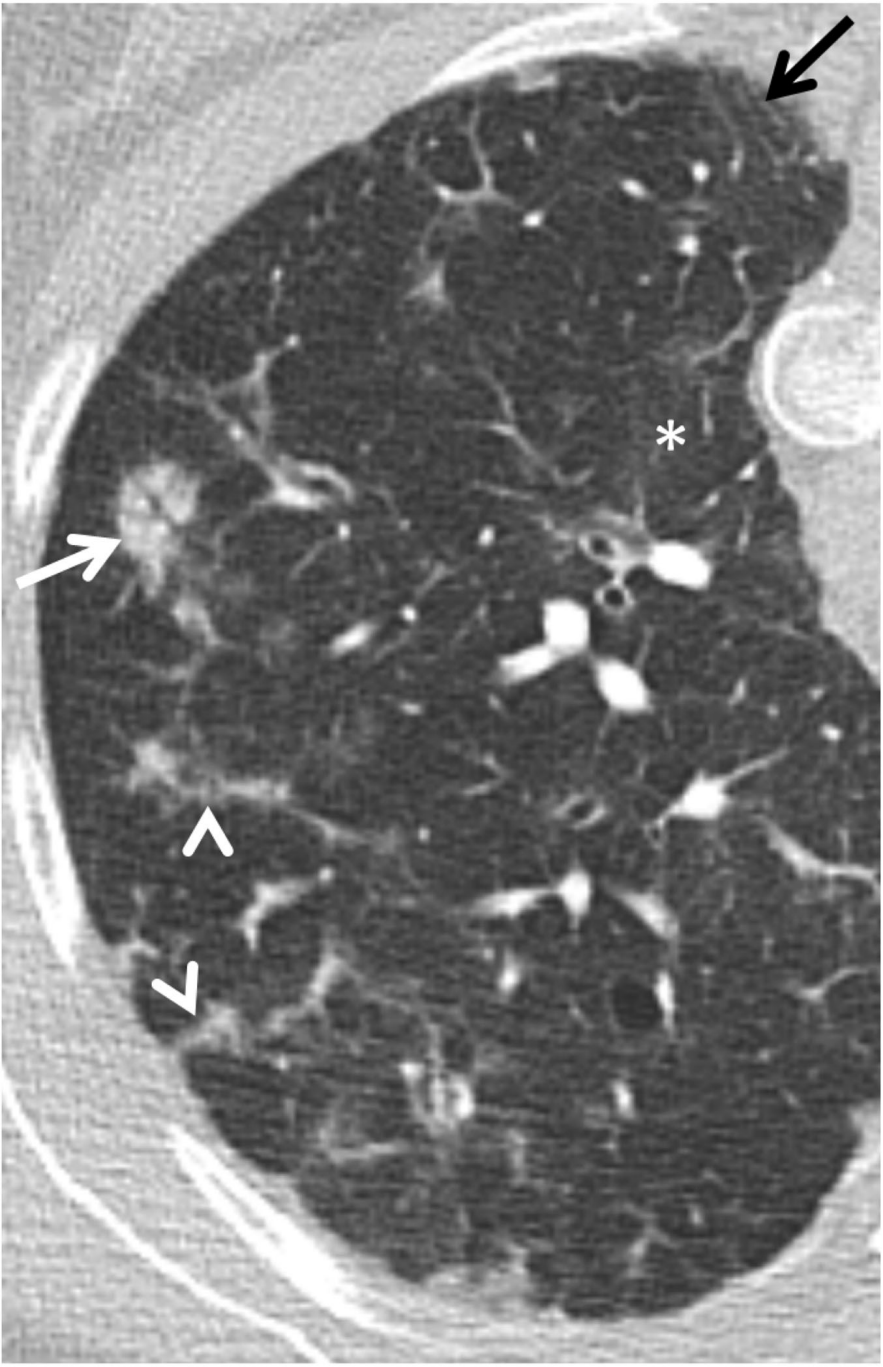
HRCT image of the right upper lobe of a 40-year-old woman with characteristic findings of granulomatous-lymphocytic interstitial lung disease (GLILD) with irregular peribronchovascular interstitial thickening (white arrow), interlobular septal thickening (arrowheads), subtle ground glass opacities (asterix), and traction bronchiectasis (black arrow).

The presence, extent, and distribution of ILD were evaluated. According to the CT criteria of ILD recommended by the Nomenclature Committee of the Fleischner Society, ILD findings include groundglass opacity, airspace consolidation, reticular patterns, and interlobular septal thickening (21), see **Figure 1**. The presence of associated findings was also assessed, such as bronchiectasis and bronchiolectasis, nodules and micronodules, thickening of peribronchovascular interstitium, pleural irregularity, mosaic attenuation pattern, mucus plugging, and air trapping. Subsegmental air trapping comprising less than 5% of the lung parenchyma was considered normal (22). CT detected ILD was defined as reticular pattern; and/or ground glass opacities, and/or consolidations; and/or nodules (except centrilobular distributed micronodules); and/or traction bronchiectasis, whereas CT detected airways disease was defined as bronchiectasis; and/or air trapping; and/or mosaic pattern; and/or centrilobular micronodules.

The extent of ground glass opacities and consolidation in each segment was assigned a score based on the percentage of lung parenchyma involved (0, no involvement; 1, 1 to 4% involvement; 2, 5 to 20% involvement; and 3, more than 20% involvement). The severity of traction bronchiectasis was scored 0–3 (1=bronchial wall thickening without distinct ectasias; 2, mild or moderate; and 3, severe bronchiectasis). Nodules, interlobular septal thickening and peribronchovascular interstitial thickening were scored 0–3 (0, absent; 1, mild; 2, moderate; and 3, severe). An overall score of abnormality involvement for each patient was derived by summing the scores of the 18 segments for each finding. Thus, both the overall extent of lung disease (regardless of pattern) and the extent of individual findings were scored, using approximately 45 min evaluating each CT scan.

### 18F-2-Fluoro-2-Deoxy-D-Glucose PET/CT Imaging

Seventeen patients underwent 18F-2-fluoro-2-deoxy-D-glucose (^18^F-FDG) positron emission tomography/CT (PET/CT) at our center during the follow-up period. In patients where PET/CT was performed more than once, the most recent was chosen. Three of the nine patients treated with rituximab for GLILD were examined with PET/CT before and after treatment, and these images were compared.

All PET/CT procedures were performed according to the European Association of Nuclear Medicine (EANM) guidelines to ensure comparability between patients, which include quality control, calibration, and harmonization of the scanners and SUV calculations and the PET/CT scans were performed on EARL-accredited (EANM Research Ltd) PET/CT systems GE Discovery 690 (n=16) and [Siemens Biograph 64 (n=5)] (23). The patients fasted for at least 6 h, and blood samples were obtained to document blood glucose levels (median 5.0 mmol/L, range 4.2–9.1 mmol/L) prior to intravenous administration of median 186 MBq ^18^F-FDG (range 120–296 MBq) and median 370 MBq ^18^F-FDG (range 233–404 MBq) for the GE Discovery and Siemens Biograph scanners, respectively. Images were obtained approximately 60–90 min. post-injection (median 70, range 63-115 min). A low-dose CT scan was performed and followed by a 3D PET scan using a whole-body acquisition protocol from the vertex to below the knee. PET acquisition times were 2.5 min/field of view (FOV) for the GE Discovery scanner and 3 min/FOV Siemens Biograph scanner.

### Quantitative PET Image Evaluation

The primary analysis of the ^18^F-FDG PET/CT images was conducted by individual image evaluation using PMOD software (PMOD Technologies LLC, version 3.510). To obtain regions of interest (ROI) in the lung, transverse slices of the fused PET/CT images were manually contoured from the apex to the base of both lungs (slice thickness of 2.79mm). Surrounding structures, including hilar regions, were excluded. The mean standardized uptake value (SUVmean), the maximum standardized uptake value (SUVmax), and lung volumes were calculated by the software. An adaptive thresholding algorithm defining a threshold of 41% of the SUVmax–SUVmin measured the metabolic lung volume (MLV) (24, 25). Total lung glycolysis (TLG) was calculated by multiplying MLV with SUVmean of MLV.

### Statistics

Associations between stable or progressive GLILD and categorical clinical parameters were assessed by chi square tests. Differences in continuous variables between two groups were analyzed using non-parametric Mann Whitney tests. Paired samples were analyzed using the Wilcoxon rank sum test. Changes in DLCO and FVC before and after treatment with rituximab were analyzed comparing the last value before the first treatment with the best available value after treatment. Annual rate of change in percent predicted DLCO was calculated by linear regression analysis. Kruskal-Wallis test was used to analyze differences between more than two groups. All tests were two-sided with a significance level of 0.05.

## Results

### Patient Characteristics

We identified 35 patients with CTs suggestive of ILD. After review by two chest radiologists, three of these patients were deemed not likely to have GLILD and were excluded from the study, leaving 32 patients with radiologic features consistent with GLILD. The patients are characterized in **Table 1**. Two patients in our cohort have been diagnosed with lymphoma, diagnosed and treated after data registry for this study. Of other malignancies in this cohort, two were treated for breast cancer and one for prostate cancer. Three of the patients were deceased (at age 36, 48, and 73). Median follow-up time was 123 months (IQR 40–156). Four of the 32 patients had a possible monogenic defect with a known association to CVID, two of these patients were in the progressive group [CTLA4-haploinsufficieny not previously described variant but likely pathogenic; STAT3 variant of uncertain significance (VUS)] and two in the stable group (NFkB1 and BACH2, both VUS). Five of the patients in our cohort had lung biopsy performed, all transbronchial. Only one of these revealed granulomas; the other four showed non-specific inflammation.

**Table 1:** Patient characteristics.

	All patients (n = 32)	Stable disease (n = 13)	Progressive disease (n=19)	p-value*
Age (years)**	48 (37–59)	44 (37–56)	51 (39–61)	0.274
Female sex, n (%)	17 (53)	5 (39)	12 (63)	0.169
Known monogenic defect,*** n (%)	4 (13)	2 (15)	2 (11)	0.683
Coexisting obstructive lung disease, n (%)	4 (13)	1 (8)	3 (16)	0.496
History of smoking, n (%)	6 (19)	2 (15)	4 (21)	0.687
First DLCO at our clinic (% of predicted)**	77 (65–85)	81 (65–85)	75 (67–83)	0.828
First FVC at our clinic (% of predicted)**	96 (75–105)	99 (90–109)	82 (69–105)	0.172
Follow-up time (months)**	123 (40–156)	73 (15–74)	142 (59–157)	0.033
**Other non-infectious complications**				
Lymphadenopathy, n (%)	30 (94)	11 (85)	19 (100)	0.077
Splenomegaly, n (%)	29 (91)	12 (92)	17 (90)	0.787
CVID associated enteropathy, n (%)	14 (44)	5 (39)	9 (47)	0.618
Autoimmune cytopenia, n (%)	12 (38)	6 (46)	6 (32)	0.403
Granulomas in other tissue, n (%)	12 (38)	5 (39)	7 (37)	0.926
NRH in liver, n (%)	8 (25)	3 (23)	5 (26)	0.835
**Immunoglobulin substitution form^§^**				
IVIG, n (%)	11 (34)	2 (15)	9 (47)	0.061
SCIG, n (%)	18 (56)	7 (54)	11 (58)	0.821
fSCIG, n (%)	5 (16)	3 (23)	2 (11)	0.337
**Immunomodulatory treatment for GLILD**				
Any treatment (%)	12 (38)	2 (15)	10 (53)	0.033
Rituximab (%)	8 (25)	1 (8)	7 (37)	0.034
Corticosteroids (%)	8 (25)	2 (15)	6 (32)	0.300
Azathioprine (%)	7 (22)	0 (0)	7 (37)	0.013
Abatacept (%)	1 (3)	0 (0)	1 (5)	0.401
Anti TNF agents (%)	1 (3)	0 (0)	1 (5)	0.401
**Immunomodulatory treatment, other indications**				
Rituximab (%)	4 (13)	2 (15)	2 (11)	0.683
Corticosteroids (%)	15 (47)	7 (54)	8 (42)	0.513

*Stable and progressive disease compared.

**Median and interquartile range.

***Whole exome sequencing performed in 29/32 patients.

^§^IVIG, intravenous immunoglobulins; SCIG, subcutaneous immunoglobulins; fSCIG, fascilitated SCIG.

### Stable and Progressive Clinical Disease

Nineteen patients (59%) were found to have progressive GLILD and 13 (41%) to have stable GLILD. The stable and the progressive group were similar with respect to gender, age, history of smoking, and co-existing obstructive lung disease. The median follow-up time, however, was shorter in the stable than the progressive group (73 *vs*. 142 months, respectively. p=0.033). Importantly, we found no significant difference in initial FVC or DLCO between patients who later developed progressive versus stable disease.

### Co-Existing Non-Infectious Complications

The majority of GLILD patients had splenomegaly (91%) and lymphadenopathy (94%). Also, a considerable proportion had had autoimmune cytopenias (38%), 25% had liver disease with biopsy verified nodular regenerative hyperplasia (NRH), 44% had biopsy verified CVID associated enteropathy, and 38% had granulomas in other tissue. We found no difference in the prevalence of co-existing non-infectious complications between the stable and the progressive GLILD group.

### Immunological Parameters

The median fraction of class-switched B-cells and plasmablasts in our total GLILD-cohort were 0.8% (normal range 4.3–23.0%), and 0.0% (normal range 0.3–5.1%), respectively (**Table 2**). The median fraction of CD21^low^ B-cells was 19.1% (normal range 1.2–9.4%). Four patients had a fraction of class-switched B-cells > 70% of lower limit of normal range. Patients were overall adequately substituted with immunoglobulins with median serum IgG concentration at 8.75 g/L. Twenty-seven of the 32 patients had not detectable levels of IgA. There were no differences in T- or B-cell subpopulation proportions, nor differences in IgG-, IgA-, or IgM-levels between the stable and progressive GLILD group. Also, the change in IgM-levels from the time point of the first PFT performed at our center to the last, or the last before GLILD directed therapy in patients receiving this, was not significantly different in the two groups.

**Table 2:** Laboratory data.

T- and B-cells with subpopulations*				
	Normal range	All patients (n = 32)	Stable disease (n = 13)	Progressive disease (n=19)
Total T-cells (x 10^6^/L)	800–2,400	1120 (724–1,503)	1130 (751–1,333)	966 (722–1,554)
CD4+ T-cells (x 10^6^/L)	500–1,400	554 (376–729)	555 (409–748)	553 (296–721)
CD8+ T-cells (x 10^6^/L)	200–1,000	465 (252–796)	498 (257–691)	365 (238–903)
% Follicular CD4+ T-cells	6.2–18.0	24.4 (17.3–31.4)	24.1 (17.5–29.7)	24.7 (17.1–36.0)
% Naive CD4+ T-cells	25.0–71.0	21.0 (12.5–31.8)	22.0 (16.0–30.2)	20.6 (11.6–35.4)
% Naive CD8+ T-cells	34.0–87.0	30.2 (17.5–41.1)	28.5 (15.–34.8)	33.2 (17.9–43.3)
% CD8+ early effector T-cells	2.9–16.0	15.5 (10.9–23.4)	12.0 (10.9–40.8)	18.9 (9.8–52.5)
% CD8+ late effector T-cells	2.6–58.0	49.5 (26.0–67.0)	58.7 (31.1–71.0)	41.3 (25.0–67.0)
% T_reg_	2.5–5.8	2.8 (2.0–3.6)	2.5 (1.9–3.2)	3.0 (2.1–4.0)
Total B-cells (x 10^6^/L)	100–500	90 (20–225)	107 (15–345)	66 (24–195)
% Class switched B-cells**	4.3–23.0	0.8 (0.5–1.7) *(n =27)*	0.7 (0.5–1.3) *(n = 10)*	0.8 (0.3–2.6) *(n = 17)*
% Transitional B-cells**	0.6–4.6	5.3 (2.1–12.9) *(n = 27)*	6.0 (4.0–14.0) *(n = 10)*	4.7 (2.0–12.8) *(n = 17)*
% Plasmablasts**	0.3–5.1	0.0 (0.0–0.0) *(n = 27)*	0.0 (0–0) *(n = 10)*	0.0 (0–0.05) *(n = 17)*
% CD21_low_ B-cells**	1.2–9.4	19.1 (8.9–36.6) *(n = 26)*	15.35 (8.3–27.8) *(n= 10)*	21.5 (11.8–41.4) *(n = 16)*
Immunoglobulin levels*				
IgG (g/L)	6.1–14.9	8.75 (7.53–10.05)	9.20 (6.25–10.45)	8.70 (7.60–9.30)
IgM (g/L)	0.7–4.3	0.15 (0.00–0.44)	0.12(0.00–0.30)	0.18 (0.00–1.30)
IgA (g/L)	0.4–2.1	0.00 (0.00–0.00)	0.00 (0.00–0.12)	0.00 (0.00–0.00)
ΔIgM during follow-up***		0.00 (0.00–0.34)	0.00 (−0.03–0.34)	0.00 (0.00–0.56)

*Median and interquartile range.

**Class-switched B-cells, transitional B-cells and plasmablasts were analyzed in 27 patients, CD21_low_ B-cells were analyzed in 26 patients.

***No statistically significant change in IgM between stable and progressive group.

### CT Findings

**Figure 2:**
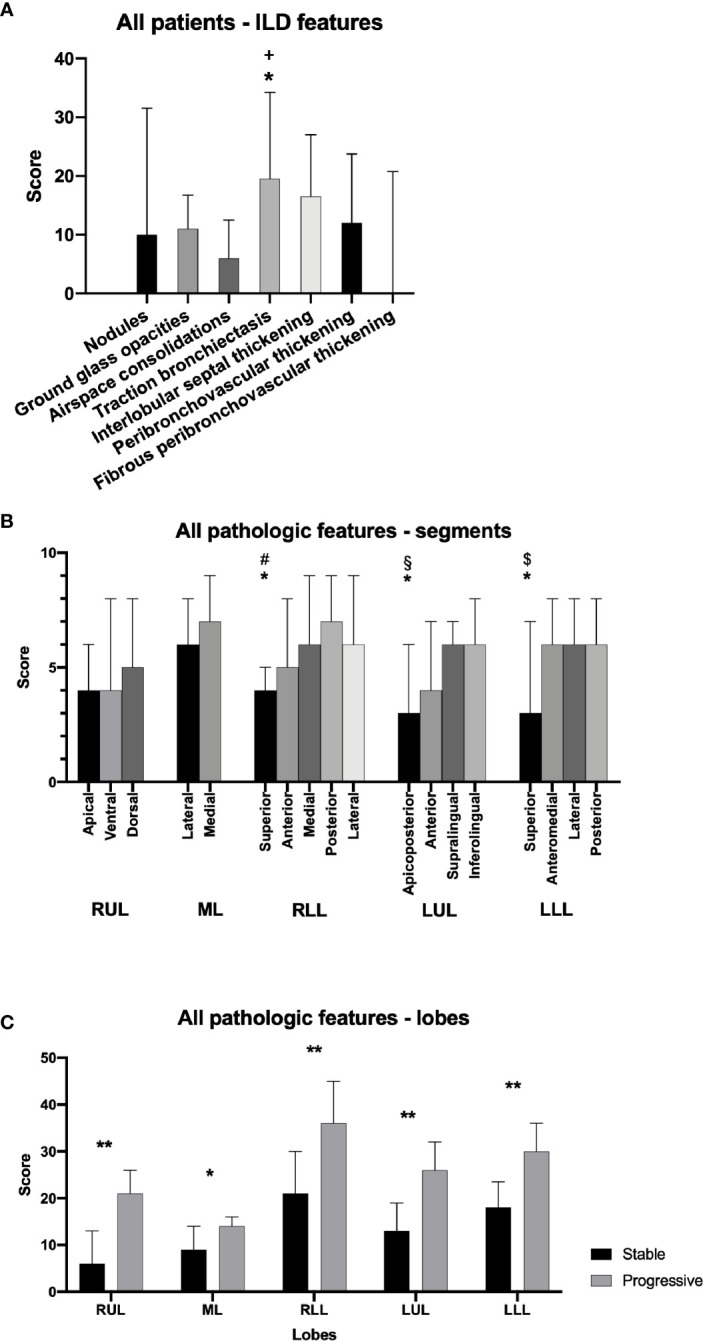
Pathologic features on pulmonary CT scans in granulomatous-lymphocytic interstitial lung disease (GLILD) patients. Overall score for specific features in all patients **(A)**. Overall score of pathologic features in all pulmonary segments for all patients **(B)**. Overall score of pathological features in single lobes in patients with stable and progressive disease **(C)**. RUL, right upper lobe; ML, middle lobe; RLL, right lower lobe; LUL, left upper lobe; LLL, left lower lobe. Median and interquartile range. *p<0.05. **p<0.01. ^+^traction bronchiectasis *vs*. ground glass opacities, nodules, consolidations, fibro-/peribronchovascular thickening. ^#^superior *vs*. lateral. ^§^apicoposterior *vs*. inferolingual. ^$^superior *vs*. posterior.

The most recent CT in each patient (in patients receiving rituximab the most recent CT prior to treatment) was scored. Traction bronchiectasis had the highest overall score of the predefined pathological radiological features, while interlobular septal thickening, ground glass opacities and peribronchovascular interstitial thickening were also frequent findings (**Figure 2A**). ILD-related pathology was present in all lobes and segments, with significantly lower scores in some of the apical segments as compared to basal segments (**Figure 2B**).

**Figure 3:**
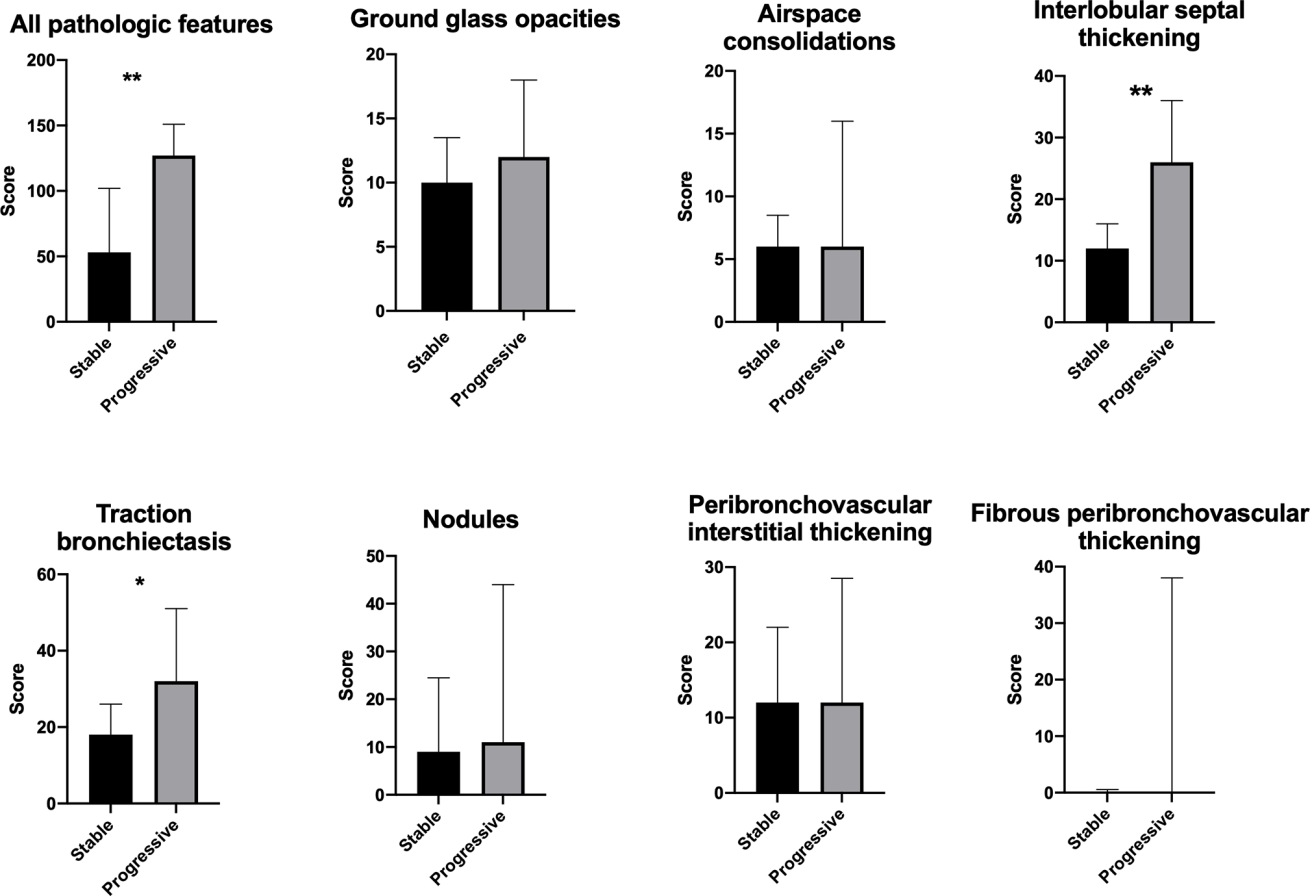
Pulmonary CT scans of patients with stable and progressive disease with score of all pathological features combined and score of specific features. Median and interquartile range. *p < 0.05. **p < 0.01.

Comparing patients with stable and progressive clinical disease, we found a significantly greater total pulmonary CT pathology in the group with progressive disease, most notably interlobular septal thickening (**Figure 3**, **Supplementary Figure 1**). Patients with progressive disease also had significantly higher score of traction bronchiectasis associated with interstitial lung disease than patients with stable disease. In addition, patients with progressive disease had increased features of overall pulmonary CT pathology in all lobes compared to patients with stable disease (**Figure 2C**). In contrast, we could not detect significant differences in scores of the specific features: ground glass opacities, airspace consolidations, nodules, peribronchovascular and fibrous peribronchovascular interstitial thickening between patients with stable and progressive clinical disease. ROC analyses showed that a threshold of 100 had a sensitivity and specificity for predicting progressive disease at 0.64 and 0.71, respectively (**Supplementary Figure 2**).

Omitting data on the four patients with possible monogenic disease did not significantly alter these CT-findings, with the exception of traction bronchiectasis that no longer differed between the stable and progressive group (**Supplementary Figures 3** and **4**).

### PET/CT Findings

**Figure 4:**
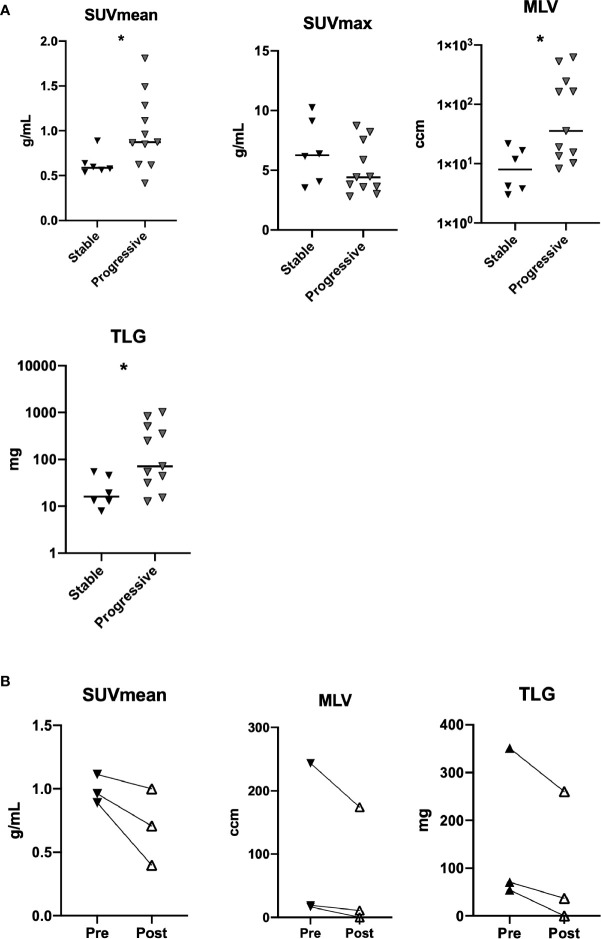
FDG PET-CT in patients with stable and progressive disease as evaluated by SUVmean, SUVmax, metabolic lung volume (MLV), and total lung glycolysis (TLG) (n=17) **(A)**. SUVmean, MLV, and TLG in patients before and after treatment with rituximab (n=3) **(B)**. *p < 0.05.

^18^F-FDG PET/CT was performed in a subgroup of the GLILD cohort with six patients with stable and eleven patients with progressive disease. Patients with progressive disease had significantly higher SUVmean in the lungs as compared to patients with stable disease (**Figure 4A**). A similar pattern was seen for MLV and TLG, while SUVmax did not significantly differ between the two patient groups. Omitting data on patients with possible monogenic disease the above-mentioned differences were non-significant (**Supplementary Figure 3**).

### Immunomodulatory Treatment

Twelve (37.5%) of our patients had received immunomodulatory treatment targeting GLILD at any time while followed at our clinic. Nine patients had been treated with rituximab, six with prednisolone, seven with azathioprine, one with abatacept, and one with adalimumab. Four patients received rituximab and 15 patients were treated with corticosteroids for other inflammatory complications than GLILD during follow up (**Table 1**).

The nine patients treated with rituximab targeting GLILD received two infusions of 1 g rituximab intravenously 2 weeks apart, every 6 months depending on treatment response. The rituximab treatment was given as monotherapy in two patients, and was combined with 100–200 mg azathioprine in seven patients, however two of these discontinued azathioprine within the three first months. Four of the seven patients that received azathioprine also received a small dose of prednisolone (5–10 mg). Eight of the nine patients treated with rituximab classified as having progressive disease.

**Figure 5:**
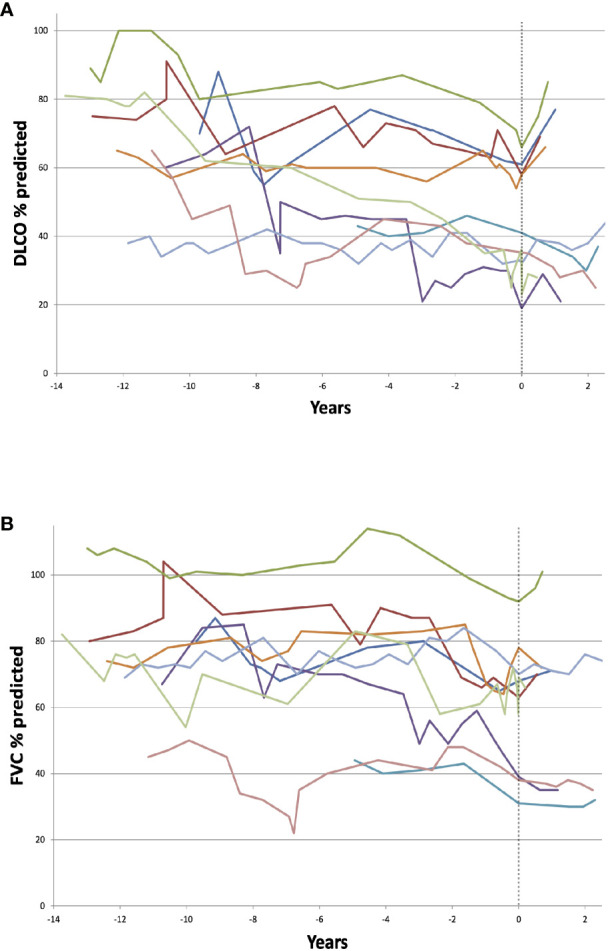
Timeline of diffusion capacity of carbon monoxide (DLCO) **(A)** and FVC **(B)** in nine individual granulomatous-lymphocytic interstitial lung disease (GLILD)-patients treated with rituximab. Dotted line represents time of first rituximab treatment. There was no significant change after treatment in % predicted DLCO or forced vital capacity (FVC). The four patients with a more preserved pre-treatment DLCO with (> 55% of predicted) had a higher annual rate of increase in percent predicted DLCO after treatment than the rest (p=0.016).

Longitudinal measurements of DLCO and FVC for the patients treated with rituximab are shown in **Figure 5**. We found a significant fall in both DLCO and FVC prior to treatment with rituximab (p=0.004 and p=0.004, respectively). Overall, for the nine patients treated with rituximab, there was no significant change after treatment in % predicted DLCO or % predicted FVC. Four patients had a more preserved pre-treatment DLCO with respect to the established ILD-GAP risk stratification model, namely > 55% of predicted. These four patients had a higher annual rate of increase in percent predicted DLCO after treatment than the five with more impaired DLCO (p=0.016). We did not find any effect of rituximab treatment on levels of CD3+, CD4+, or CD8+ lymphocytes, nor levels of IgM or IgA (data not shown).

**Figure 6:**
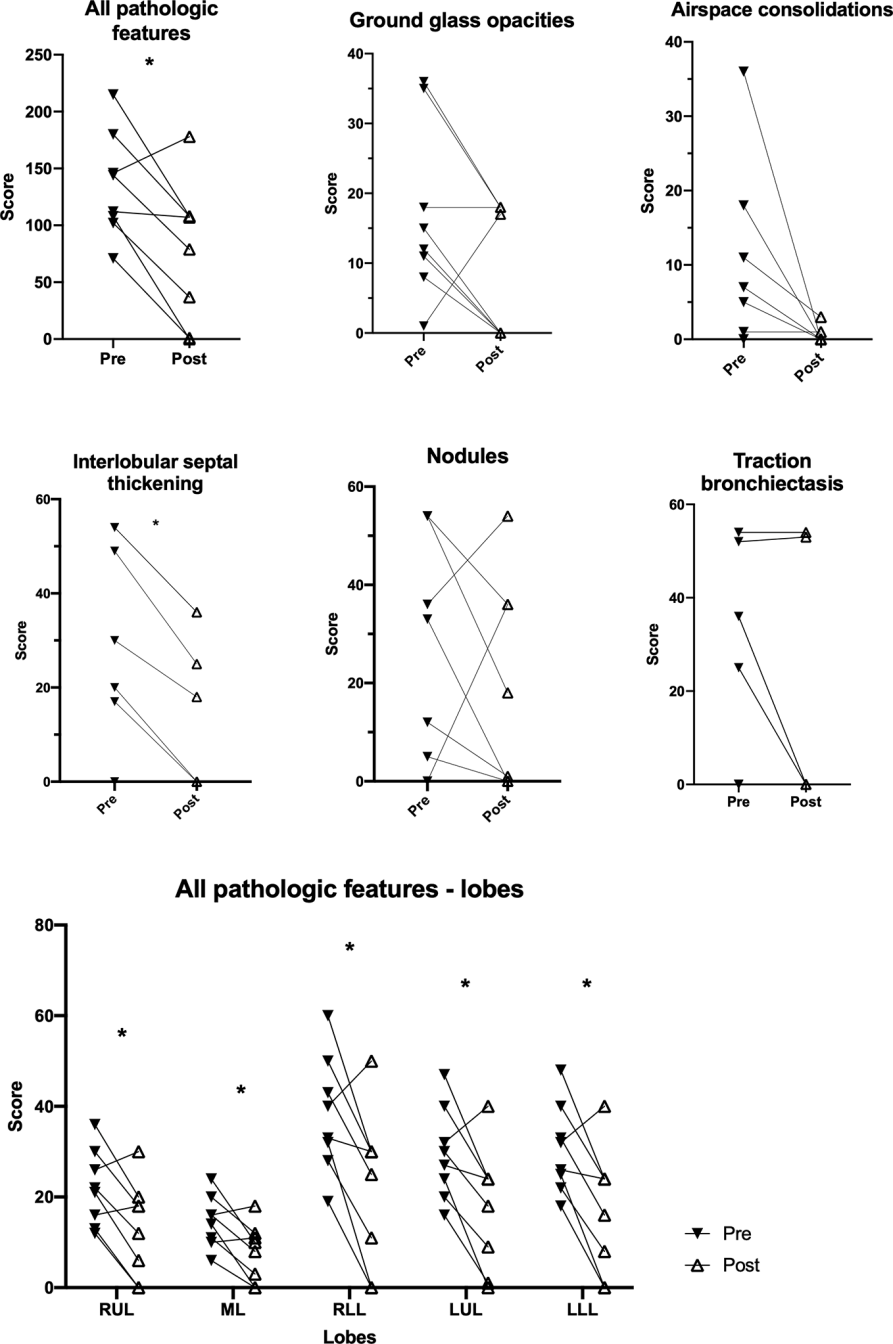
Change in pathological features on pulmonary CT scans in granulomatous-lymphocytic interstitial lung disease (GLILD) patients before and after treatment with rituximab. RUL, right upper lobe; ML, middle lobe; RLL, right lower lobe; LUL, left upper lobe; LLL, left lower lobe. Median and interquartile range. *p < 0.05.

CT scans performed 6–18 months after the initial dose of rituximab were scored and compared to the most recent pretreatment CT (available in eight patients). We found a significant reduction in overall pulmonary pathology after rituximab treatment, and this improvement was present in all lobes (**Figure 6**). Comparing the extent of the ILD specific radiological features separately before and after treatment, with the exception of interlobular septal thickening changes in each of these were not significant (changes in peribronchovascular interstitial thickening and fibrous peribronchovascular interstitial thickening not shown) (**Figure 6**). Omitting data on patients with possible monogenic disease did not significantly alter these findings (**Supplementary Figure 6**).

**Figure 7:**
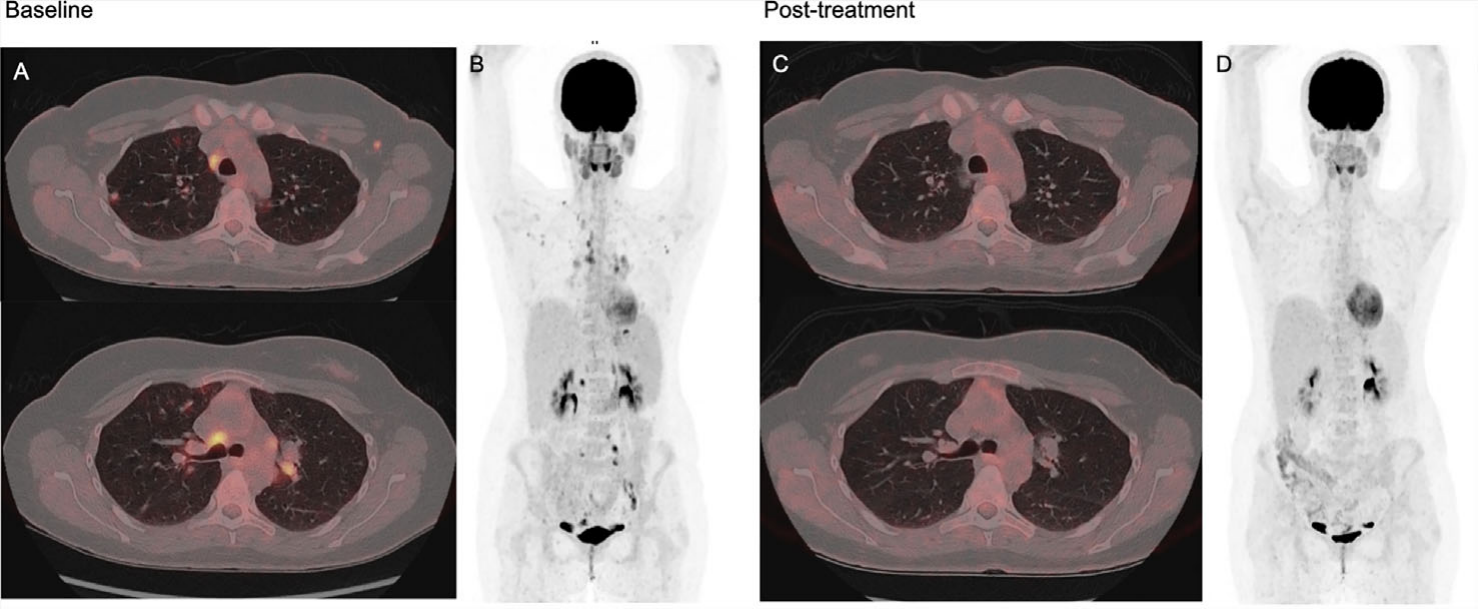
FDG PET/CT at baseline and 3–4 months after monotherapy with rituximab in a 37-year-old common variable immunodeficiency (CVID) patient with granulomatous-lymphocytic interstitial lung disease (GLILD) and generalized lymphadenopathy. Image **(A)** shows axial fused PET/CT images at two different thoracic levels at baseline, with scattered nodular and confluent consolidations in the pulmonary parenchyma with moderate to high FDG uptake, and also moderate to high FDG uptake in mediastinal and hilar lymph nodes. Image **(B)** shows baseline maximum intensity projection (MIP) showing pathologic FDG uptake in lung parenchyma and lymph nodes over and under the diaphragm. Image **(C, D)** shows post-treatment axial fused FDG PET/CT and MIP with complete resolution of both pulmonary and lymph node pathology. Spleen size was within normal range both before and after treatment.

Three patients were evaluated with ^18^F-FDG PET/CT before and after treatment with rituximab. There was a decline in SUVmean, SUVmax, MLV, and TLG for all three patients after treatment (**Figure 4B**, data on SUVmax not shown; **Figure 7**).

## Discussion

In this retrospective study of 32 CVID patients with GLILD, we found that patients with clinical progression based on pulmonary functional tests had a significantly greater extent of ILD features on thoracic CT, and more prominent pulmonary inflammation in ^18^F-FDG PET/CT than those with stable clinical disease. Most notably, patients with progressive clinical disease had a greater extent of traction bronchiectasis and interlobular septal thickening.

In our cohort, 19 of 32 patients had progressive clinical disease, comparable to another previously described cohort (26). Progressive disease can be defined by an absolute decline in pulmonary function but also by decline per time, and we have used the former definition in our study. However, existing data on non-invasive parameters associated with clinical disease progression are scarce. Herein we show that the systematic scoring of pulmonary pathology on CT scans and ^18^F-FDG PET/CT characteristics could be important diagnostic tools when evaluating disease progression and treatment response in CVID patients with GLILD.

Histopathological features of GLILD may include features of LIP and follicular bronchiolitis (4, 6). Typical CT findings of LIP include ground-glass opacities, bronchovascular bundle thickening (which is similar to peribronchovascular interstitial thickening in our study), and mild interlobular septal thickening, which are overlapping with the CT findings in our GLILD cohort (5, 11, 27). Several GLILD patients had architectural remodeling with traction bronchiectasis, which is a typical finding in ILD and, notably, the presence of this finding was significantly higher in the patients with clinical progression of GLILD. Moreover, interlobular septal thickening and traction bronchiectasis discriminated most clearly between those with and without clinical progression. In contrast, several other features of both LIP and follicular bronchiolitis such as cysts, poorly defined centrilobular nodules and small subpleural nodules, were uncommon findings in our patients. Likewise, intralobular reticular patterns and honeycombing typically seen in fibrotic non-specific interstitial pneumonia (NSIP) and unspecific interstitial pneumonia (UIP) were not identified. These findings may suggest that ILD in CVID patients has other characteristics, and potentially also represents different pathophysiological mechanisms than ILD in patients without underlying immunodeficiency. However, these important issues will have to be studied in larger prospective cohorts of CVID patients with GLILD.

Previous data on the use of ^18^F-FDG PET/CT in evaluating GLILD in CVID patients are scarce, but our data suggest that this could be a valuable tool in the management of GLILD. Indeed, our data showed a significantly higher SUVmean, MLV, and TLG in patients with progressive disease. The SUVmean and volume based MLV and TLG have recently shown to be better prognostic indicators than SUVmax in several studies (28, 29). SUVmax represents the value from one single voxel and does not quantify the total inflammatory burden such as SUVmean, MLV, and TLG (25). Furthermore, a single SUVmax measurement can be unreliable, especially when glucose uptake is heterogeneous and the disease is systemic with multiple lesions such as in GLILD. Thus, SUVmean, MLV, and TLG can provide sensitive and specific values that give insight to the stage and progression of the disease. ^18^F-FDG PET/CT could therefore be used to identify patients with active pulmonary inflammation and progressive disease, as well as evaluate therapeutic measures with a quantitative analysis. In this study we focused on ^18^F-FDG PET/CT imaging of the lungs only. However, a measurement of the total inflammatory burden, by total body FDG uptake in these patients would be of interest, and subject for future studies.

In contrast to CT and FDG PET-CT, magnetic resonance imaging (MRI) have the advantage of using non-ionizing radiation but has not been systematically evaluated for follow-up of interstitial lung disease (30).

Rituximab has emerged as a preferred second-line treatment for GLILD in combination with immunomodulatory agents. In this retrospective study we included nine patients that were treated with rituximab. As others have reported, overall pulmonary pathology on CT improved clearly after treatment with rituximab (7, 14, 15, 17). There was a generalized pattern of improvement in all lobes, but no change in specific features reached statistical significance, possibly due to low number of patients treated. Furthermore, treatment with rituximab alone or in combination with azathioprine or mycophenolate has been shown to improve functional tests such as FVC and DLCO (7, 13, 15–17). In our nine patients, we did not find any significant change in either DLCO or FVC after rituximab treatment, but the subgroup of four patients with a relatively preserved pre-treatment DLCO (> 55% predicted), showed a greater annual increase in percent predicted DLCO than the remaining five with lower pre-treatment DLCO. This heterogeneity and the small number of patients may explain the discrepancy between changes in CT and PFT. The question of when to start treatment of GLILD is difficult and unanswered, but this observation argues for early initiation of treatment. However, the small number of patients here does not allow for any absolute conclusions.

Patients had similar levels of IgG after substitution and comparable substitution regimens, suggesting that the mode of immunoglobulin substitution has no major influence on GLILD progression, even if it has been claimed that IVIG has immunomodulatory properties that could be beneficial in inflammatory complications of CVID.

Considering the lack of a universally accepted definition of CVID, for the purpose of this study, we found it appropriate to use a broad definition to include patients that we recognize, monitor and treat as CVID with GLILD (2, 31). Four of the patients in our cohort did not fulfill the ESID 2019 definition of smB-cells < 70% of lower limit of normal range (32). We did not have documentation of poor vaccine antibody response in these. All the patients in our cohort had low levels of IgA, and the other “ESID 2019” criteria were met to fulfil the diagnosis.

The present study has several limitations such as its retrospective nature. The lack of longitudinal data on most of the parameters, a low number of patients in the observational rituximab sub-study and a relatively short follow-up time after rituximab treatment are also important limitations. The follow-up time was shorter in the stable group, limiting this study since the definition of progression is partly dependent on observation time. However, the fact that the age of the patients in the two groups were similar, and that we also included patients with pathological pulmonary function tests at first visit at our center is a compensating factor. The data from the patients treated with rituximab should be interpreted with caution based on the low number of patients and the retrospective observational design of the study. CT scans were evaluated qualitatively and even if this were by independent experienced radiologists the lack of quantitative analyses is a limitation of the study. The lack of exercise tolerance test data, data on self-reported dyspnea and frequency of airway infections in this cohort are further limitations of this study. Four of the patients had possible monogenic defects, including one patient with a likely CTLA4-haploinsufficiency, but these were evenly distributed in the two groups.

## Conclusion

In this study of 32 CVID-patients with radiological features consistent with GLILD, we found that a majority of patients had progressive disease defined by a decline in PFT results over time. We found a significantly higher overall CT pathology score in patients with progressive GLILD compared to patients with stable GLILD, with interlobular septal thickening and traction bronchiectasis as the most prominent findings. Patients with progressive disease furthermore had significantly higher SUVmean, MLV, and TLG on FDG-PET/CT suggesting that this modality may be valuable for identifying patients with active pulmonary inflammation and progressive disease, thus complementing CT as a tool in the evaluation of when to start treatment for GLILD. In our cohort, treatment with rituximab was followed by a significant improvement in overall pulmonary CT pathology, while changes in pulmonary function varied. GLILD remains a significant clinical challenge, and identifying factors contributing to disease progression and to clinical improvement following treatment will be important to improve care for these patients.

## Data Availability Statement

The raw data supporting the conclusions of this article will be made available by the authors, without undue reservation.

## Ethics Statement

The studies involving human participants were reviewed and approved by REC South-Eastern Norway. The patients/participants provided their written informed consent to participate in this study.

## Author Contributions

MSAF, NM, MR, MTD, IN, MEM, PA, SFJ, TMA, and BF designed the study. MSAF, NM, MR, MLS, TMA, and BF analyzed the data. All authors contributed to the writing of the manuscript and read the final version. All authors contributed to the article and approved the submitted version. The publication of this article was made possible through funding from the Norwegian Immunodeficiency Society.

## Funding

The publication of the article was made possible through funding from the Norwegian Immunodeficency Society. 

## Conflict of Interest

The authors declare that the research was conducted in the absence of any commercial or financial relationships that could be construed as a potential conflict of interest.

## References

[1] Cunningham-Rundles C How I treat common variable immune deficiency. Blood (2010) 116(1):7–15. 10.1182/blood-2010-01-254417 20332369 PMC2904582

[2] Bonilla FA Barlan I Chapel H Costa-Carvalho BT Cunningham-Rundles C de la Morena MT International Consensus Document (ICON): Common Variable Immunodeficiency Disorders. J Allergy Clin Immunol Pract (2016) 4(1):38–59. 10.1016/j.jaip.2015.07.025 26563668 PMC4869529

[3] Ho HE Cunningham-Rundles C Non-infectious Complications of Common Variable Immunodeficiency: Updated Clinical Spectrum, Sequelae, and Insights to Pathogenesis. Front Immunol (2020) 11:149. 10.3389/fimmu.2020.00149 32117289 PMC7025475

[4] Bates CA Ellison MC Lynch DA Cool CD Brown KK Routes JM Granulomatous-lymphocytic lung disease shortens survival in common variable immunodeficiency. J Allergy Clin Immunol (2004) 114(2):415–21. 10.1016/j.jaci.2004.05.057 15316526

[5] Rao N Mackinnon AC Routes JM Granulomatous and lymphocytic interstitial lung disease: a spectrum of pulmonary histopathologic lesions in common variable immunodeficiency–histologic and immunohistochemical analyses of 16 cases. Hum Pathol (2015) 46(9):1306–14. 10.1016/j.humpath.2015.05.011 PMC4554947 26138782

[6] Larsen BT Smith ML Tazelaar HD Yi ES Ryu JH Churg A GLILD Revisited: Pulmonary Pathology of Common Variable and Selective IgA Immunodeficiency. Am J Surg Pathol (2020) 44(8):1073–81. 10.1097/PAS.0000000000001479 32235152

[7] Verbsky JW Hintermeyer MK Simpson PM Feng M Barbeau J Rao N Rituximab and antimetabolite treatment of granulomatous and lymphocytic interstitial lung disease in common variable immunodeficiency. J Allergy Clin Immunol (2020) S0091-6749(20)31069-1. 10.1016/j.jaci.2020.07.021 32745555

[8] Schussler E Beasley MB Maglione PJ Lung Disease in Primary Antibody Deficiencies. J Allergy Clin Immunol Pract (2016) 4(6):1039–52. 10.1016/j.jaip.2016.08.005 PMC5129846 27836055

[9] Maglione PJ Overbey JR Radigan L Bagiella E Cunningham-Rundles C Pulmonary radiologic findings in common variable immunodeficiency: clinical and immunological correlations. Ann Allergy Asthma Immunol (2014) 113(4):452–9. 10.1016/j.anai.2014.04.024 PMC4177442 24880814

[10] Park JE Beal I Dilworth JP Tormey V Haddock J The HRCT appearances of granulomatous pulmonary disease in common variable immune deficiency. Eur J Radiol (2005) 54(3):359–64. 10.1016/j.ejrad.2004.09.005 15899336

[11] Tanaka N Kim JS Bates CA Brown KK Cool CD Newell JD Lung diseases in patients with common variable immunodeficiency: chest radiographic, and computed tomographic findings. J Comput Assisted Tomography (2006) 30(5):828–38. 10.1097/01.rct.0000228163.08968.26 16954938

[12] Jolles S Carne E Brouns M El-Shanawany T Williams P Marshall C FDG PET-CT imaging of therapeutic response in granulomatous lymphocytic interstitial lung disease (GLILD) in common variable immunodeficiency (CVID). Clin Exp Immunol (2017) 187(1):138–45. 10.1111/cei.12856 PMC5167039 27896807

[13] Zdziarski P Gamian A Lymphoid Interstitial Pneumonia in Common Variable Immune Deficiency - Case Report With Disease Monitoring in Various Therapeutic Options: Pleiotropic Effects of Rituximab Regimens. Front Pharmacol (2018) 9:1559. 10.3389/fphar.2018.01559 30713498 PMC6346143

[14] Chase NM Verbsky JW Hintermeyer MK Waukau JK Tomita-Mitchell A Casper JT Use of combination chemotherapy for treatment of granulomatous and lymphocytic interstitial lung disease (GLILD) in patients with common variable immunodeficiency (CVID). J Clin Immunol (2013) 33(1):30–9. 10.1007/s10875-012-9755-3 PMC3557581 22930256

[15] Ng J Wright K Alvarez M Hunninghake GM Wesemann DR Rituximab Monotherapy for Common Variable Immune Deficiency-Associated Granulomatous-Lymphocytic Interstitial Lung Disease. Chest (2019) 155(5):e117–e21. 10.1016/j.chest.2019.01.034 PMC6689079 31060706

[16] Maglione PJ Gyimesi G Cols M Radigan L Ko HM Weinberger T BAFF-driven B cell hyperplasia underlies lung disease in common variable immunodeficiency. JCI Insight (2019) 4(5):e122728. 10.1172/jci.insight.122728 PMC6483510 30843876

[17] Cereser L De Carli R Girometti R De Pellegrin A Reccardini F Frossi B Efficacy of rituximab as a single-agent therapy for the treatment of granulomatous and lymphocytic interstitial lung disease in patients with common variable immunodeficiency. J Allergy Clin Immunol Pract (2019) 7(3):1055–7.e2. 10.1016/j.jaip.2018.10.041 30408616

[18] Bucciol G Petrone A Putti MC Efficacy of mycophenolate on lung disease and autoimmunity in children with immunodeficiency. Pediatr Pulmonol (2017) 52(10):E73–e6. 10.1002/ppul.23757 28672090

[19] Boursiquot JN Gérard L Malphettes M Fieschi C Galicier L Boutboul D Granulomatous disease in CVID: retrospective analysis of clinical characteristics and treatment efficacy in a cohort of 59 patients. J Clin Immunol (2013) 33(1):84–95. 10.1007/s10875-012-9778-9 22986767

[20] Ryerson CJ Vittinghoff E Ley B Lee JS Mooney JJ Jones KD Predicting survival across chronic interstitial lung disease: the ILD-GAP model. Chest (2014) 145(4):723–8. 10.1378/chest.13-1474 24114524

[21] Hansell DM Bankier AA MacMahon H McLoud TC Müller NL Remy J Fleischner Society: glossary of terms for thoracic imaging. Radiology (2008) 246(3):697–722. 10.1148/radiol.2462070712 18195376

[22] Tanaka N Matsumoto T Miura G Emoto T Matsunaga N Ueda K Air trapping at CT: high prevalence in asymptomatic subjects with normal pulmonary function. Radiology (2003) 227(3):776–85. 10.1148/radiol.2273020352 12702825

[23] Jamar F Buscombe J Chiti A Christian PE Delbeke D Donohoe KJ EANM/SNMMI guideline for 18F-FDG use in inflammation and infection. J Nuclear Med (2013) 54(4):647–58. 10.2967/jnumed.112.112524 23359660

[24] Lasnon C Enilorac B Popotte H Aide N Impact of the EARL harmonization program on automatic delineation of metabolic active tumour volumes (MATVs). EJNMMI Res (2017) 7(1):30. 10.1186/s13550-017-0279-y 28361349 PMC5374086

[25] Im HJ Bradshaw T Solaiyappan M Cho SY Current Methods to Define Metabolic Tumor Volume in Positron Emission Tomography: Which One is Better? Nuclear Med Mol Imaging (2018) 52(1):5–15. 10.1007/s13139-017-0493-6 PMC5777960 29391907

[26] Maglione PJ Overbey JR Cunningham-Rundles C Progression of Common Variable Immunodeficiency Interstitial Lung Disease Accompanies Distinct Pulmonary and Laboratory Findings. J Allergy Clin Immunol Pract (2015) 3(6):941–50. 10.1016/j.jaip.2015.07.004 PMC4641811 26372540

[27] Prasse A Kayser G Warnatz K Common variable immunodeficiency-associated granulomatous and interstitial lung disease. Curr Opin Pulmonary Med (2013) 19(5):503–9. 10.1097/MCP.0b013e3283642c47 23880700

[28] Abdulla S Salavati A Saboury B Basu S Torigian DA Alavi A Quantitative assessment of global lung inflammation following radiation therapy using FDG PET/CT: a pilot study. Eur J Nuclear Med Mol Imaging (2014) 41(2):350–6. 10.1007/s00259-013-2579-4 24085504

[29] Høilund-Carlsen PF Edenbrandt L Alavi A Global disease score (GDS) is the name of the game! Eur J Nuclear Med Mol Imaging (2019) 46(9):1768–72. 10.1007/s00259-019-04383-8 PMC6647113 31183636

[30] Weatherley ND Eaden JA Stewart NJ Bartholmai BJ Swift AJ Bianchi SJ Experimental and quantitative imaging techniques in interstitial lung disease. Thorax (2019) 74:611–9. 10.1136/thoraxjnl-2018-211779 PMC6585263 30886067

[31] Tangye SG Al-Herz W Bousfiha A Chatila T Cunningham-Rundles C Etzioni A Human Inborn Errors of Immunity: 2019 Update on the Classification from the International Union of Immunological Societies Expert Committee. J Clin Immunol (2020) 40(1):24–64. 10.1007/s10875-019-00737-x 31953710 PMC7082301

[32] Seidel MG Kindle G Gathmann B Quinti I Buckland M van Montfrans J The European Society for Immunodeficiencies (ESID) Registry Working Definitions for the Clinical Diagnosis of Inborn Errors of Immunity. J Allergy Clin Immunol Pract (2019) 7(6):1763–70. 10.1016/j.jaip.2019.02.004 30776527

