# Supplementary material for: Granulomatous-Lymphocytic Interstitial Lung Disease in Common Variable Immunodeficiency—Features of CT and 18F-FDG Positron Emission Tomography/CT in Clinically Progressive Disease
Source: Front Immunol. 2021 Jan 26;11:617985. doi: 10.3389/fimmu.2020.617985 (PMC7874137; doi:10.3389/fimmu.2020.617985)
Supplement: Supplementary file 1 [file DataSheet_1.pdf]

Granulomatous-lymphocytic interstitial lung disease in common variable immunodeficiency – features of CT and 18-FDG PET/CT in clinically progressive disease

Supplementary figures

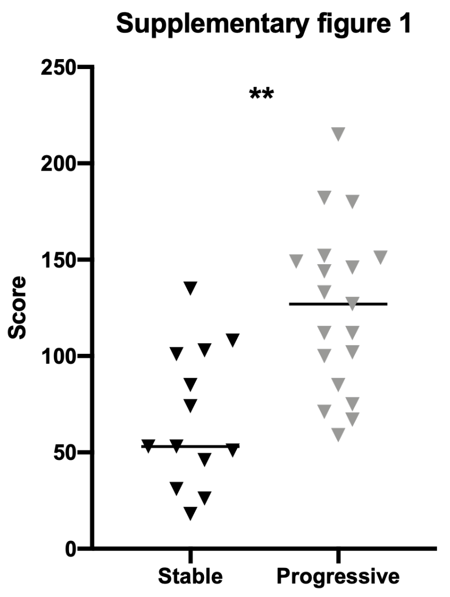

Supplementary figure legend 1. Scatter plot of overall radiologic score in patients with stable and progressive disease. **p<0.01

Supplementary figure 2

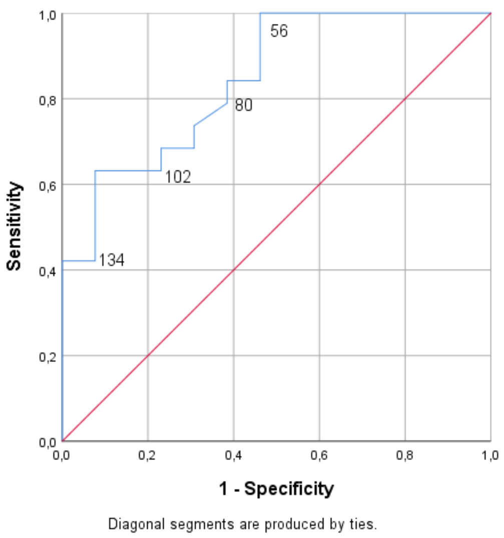

Supplementary figure legend 2. ROC analyses – all radiologic features vs progressive/stable disease. ROC analyses show good performance of the overall radiologic scoring in predicting progressive vs stable disease (AUC 0.844). A score above 63 was accompanied with a 94.7% sensitivity and 46.2% specificity for progressive disease. A score above 140 was accompanied with 42.1 % sensitivity and 100% specificity for progressive disease in our cohort. ROC

analysis performed using IBS SPSS version 25 software with clinical status as state variable and overall radiologic score as test variable.

Area Under the Curve

Test Result Variable(s): All_score_num

Area	Std. Error[a]	Asymptotic Sig.[b]	Asymptotic 95% Confidence Interval	
			Lower Bound	Upper Bound
,844	,069	,001	,709	,979

The test result variable(s): All_score_num has at least one tie between the positive actual state group and the negative actual state group. Statistics may be biased.

a. Under the nonparametric assumption

b. Null hypothesis: true area = 0.5

Coordinates of the Curve

Test Result Variable(s): All_score_num

Positive if Greater Than or Equal To[a]	Sensitivity	1 - Specificity
17,0000	1,000	1,000
22,0000	1,000	,923
28,5000	1,000	,846
38,5000	1,000	,769
48,5000	1,000	,692
52,0000	1,000	,615
56,0000	1,000	,462
63,0000	,947	,462
69,0000	,895	,462
72,5000	,842	,462
74,5000	,842	,385
80,0000	,789	,385
92,5000	,737	,308
100,5000	,684	,308
101,5000	,684	,231
102,5000	,632	,231
105,5000	,632	,154
110,0000	,632	,077
119,5000	,526	,077
130,0000	,474	,077

Coordinates of the Curve (cont.)

Positive if Greater Than or Equal To[a]	Sensitivity	1 - Specificity
134,0000	,421	,077
139,5000	,421	,000
145,0000	,368	,000
147,5000	,316	,000
150,0000	,263	,000
151,5000	,211	,000
166,0000	,158	,000
181,0000	,105	,000
198,5000	,053	,000
216,0000	,000	,000

The test result variable(s): All_score_num has at least one tie between the positive actual state group and the negative actual state group.

a. The smallest cutoff value is the minimum observed test value minus 1, and the largest cutoff value is the maximum observed test value plus 1. All the other cutoff values are the average of two consecutive ordered observed test values

Supplementary figure 3

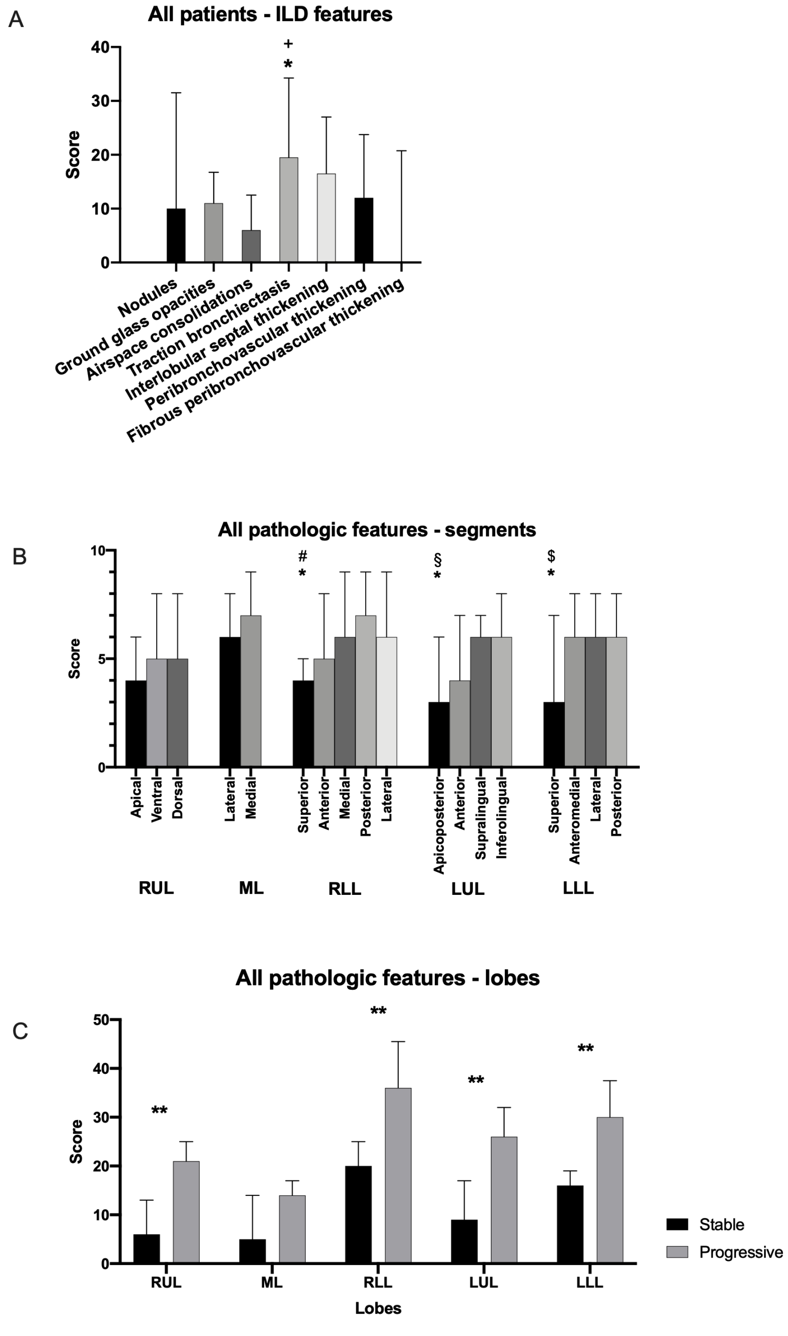

Supplementary figure legend 3 (patients without monogenic defects). Pathologic features on pulmonary CT scans in GLILD patients.Overall score for specific features in all patients (A). Overall score of pathologic features in all pulmonary segments for all patients (B). Overall score of pathological features in single lobes in patients with stable and progressive disease (C). RUL=right upper lobe. ML=middle lobe. RLL=right lower lobe. LUL=left upper lobe. LLL=left lower lobe. Median and interquartile range. *p<0.05. **p<0.01. + traction bronchiectasis vs ground glass opacities, nodules, consolidations, fibro-/peribronchovascular thickening. # superior vs lateral. § apicoposterior vs inferolingual. $ superior vs posterior.

Supplementary figure 4

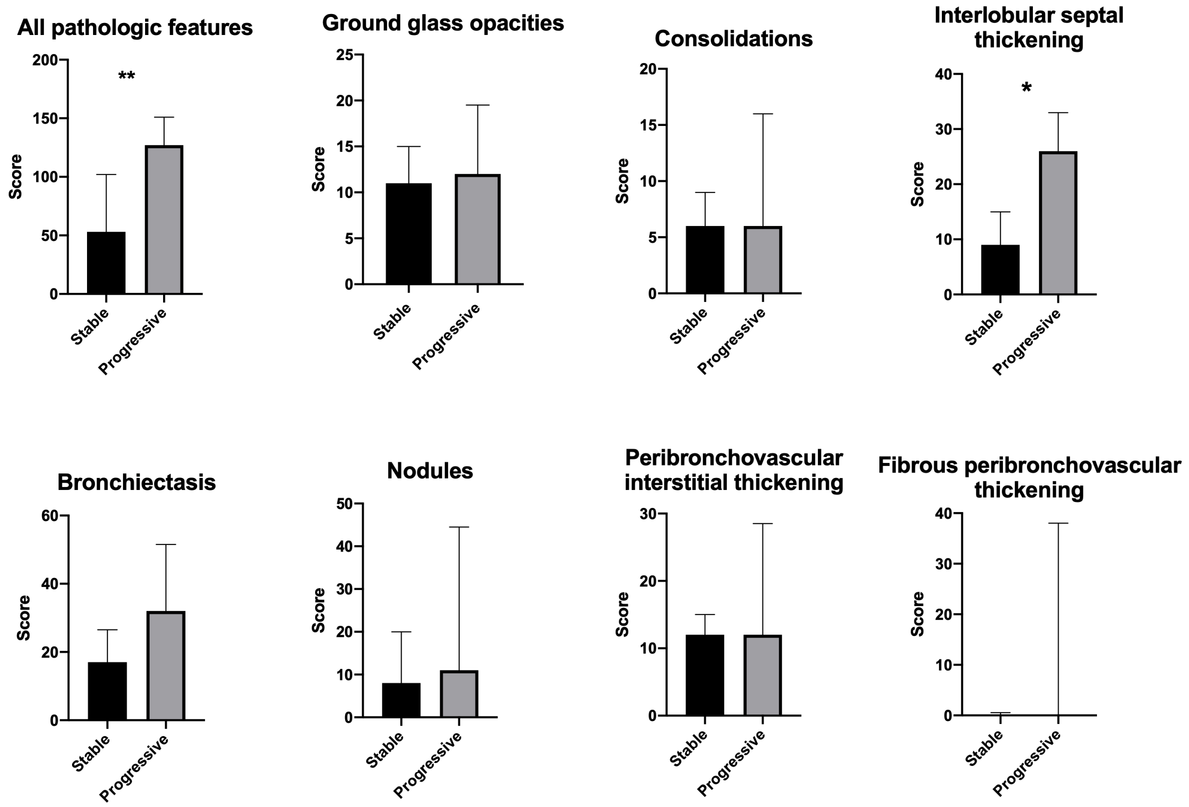

Supplementary figure legend 4 (patients without monogenic defects). Pulmonary CT scans of patients with stable and progressive disease with score of all pathological features combined and score of specific features. Median and interquartile range. *p<0.05. **p<0.01.

Supplementary figure 5

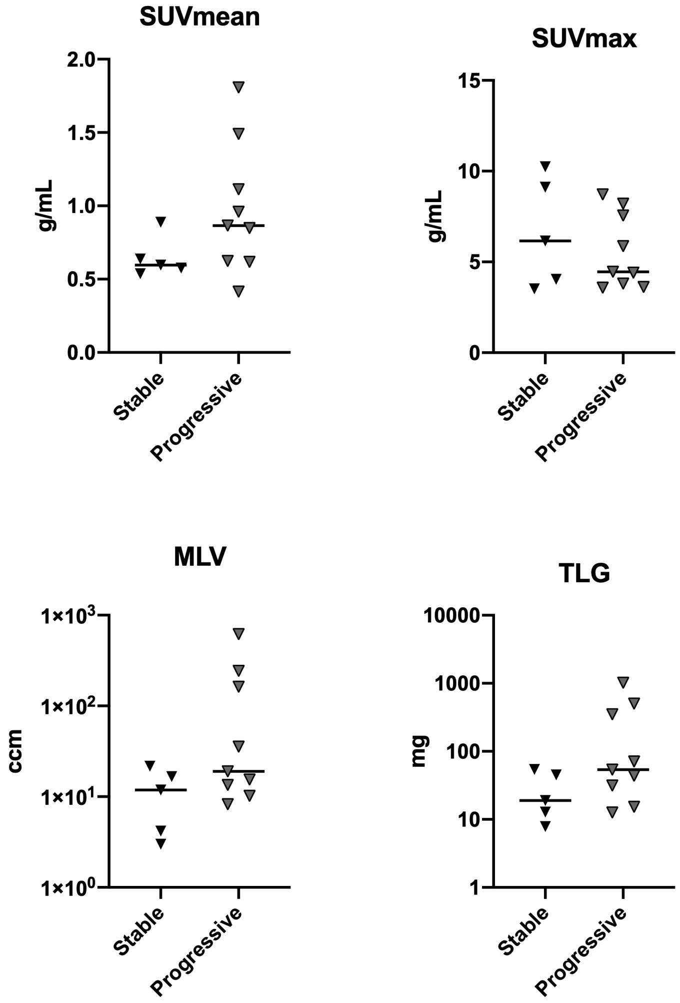

Supplementary figure legend 5 (patients without monogenic defects). FDG PET-CT in patients with stable and progressive disease as evaluated by SUVmean, SUVmax, MLV and TLG (n=17)

Supplementary figure 6

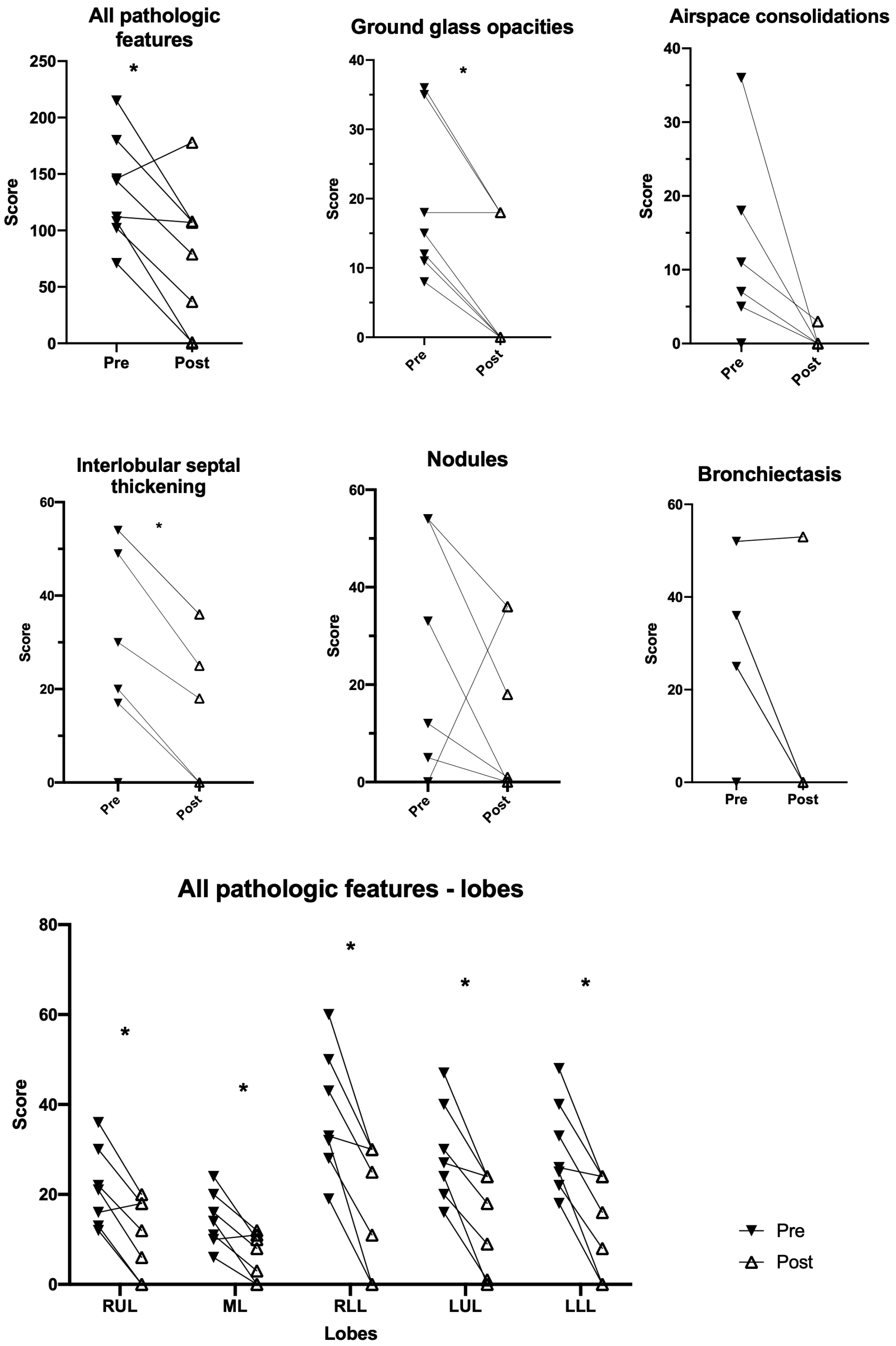

Supplementary figure legend 6 (patients without monogenic defects. Change in pathological features on pulmonary CT scans in GLILD patients before and after treatment with rituximab. RUL=right upper lobe. ML=middle lobe. RLL=right lower lobe. LUL=left upper lobe. LLL=left lower lobe. *p<0.05.
